# Leader humility and employees’ creative performance: the role of intrinsic motivation and work engagement

**DOI:** 10.3389/fpsyg.2024.1278755

**Published:** 2024-01-19

**Authors:** Haiou Liu, Syed Jameel Ahmed, Muhammad Adeel Anjum, Azalim Mina

**Affiliations:** ^1^Yanshan University, Qinhuangdao, Hebei, China; ^2^Balochistan University of Information Technology, Engineering and Management Sciences, Quetta, Balochistan, Pakistan

**Keywords:** humble leadership, creative performance, intrinsic motivation, work engagement, mediation

## Abstract

Drawing on the job demand-resource (JD-R) model and self-determination theory (SDT), this study examines the relationship between humble leadership and employees’ creative performance, taking into account the sequential mediating role of intrinsic motivation and work engagement. The sequential mediation model was tested using two-wave questionnaire data collected from employees and their supervisors (*n* = 350) in the telecommunication sector of Pakistan. Data were processed and examined using SPSS and AMOS. The results revealed significant positive relationships among all variables. Further, it was found that intrinsic motivation and work engagement sequentially but partially mediated the positive relationship between humble leadership and creative performance. The theoretical and practical implications are discussed at the end.

## 1 Introduction

The fast-paced technological changes and un-predictable working environment has made creativity crucial for organizations (Carmeli and Paulus, 2015). Creativity entails identifying new solutions to the existing problems (Amabile, 1988). Keeping in view the importance of creativity for individuals and organizations, scholars have dedicated considerable attention to identify factors that can influence it (Woods et al., 2018; De Clercq and Mustafa, 2023). However; there is a lack of emphasis on elucidating the means by which employees can excel and enhance their performance (Amabile, 1988; AlKayid et al., 2023) when working under the guidance of humble leadership (HL). Humble leadership is a bottom-up approach of acknowledging limitations and mistakes, recognizing followers’ strengths and contributions, and modeling teachability (Owens and Hekman, 2012). Humble leadership, as a concept, originates from the seminal work of Owens and Hekman (2012), who introduced it to describe a leadership style characterized by leaders acknowledging their limitations and appreciating the contributions of others. It responds to the need for leaders to navigate complex organizational landscapes by fostering collaboration and authenticity. Humble leadership encompasses behaviors such as admitting mistakes, valuing team members’ strengths, and maintaining a collective focus on success (Owens and Hekman, 2016). Recent conceptualizations highlight dimensions including self-awareness, appreciation of others, and a commitment to creating a collaborative and inclusive work environment (Ou et al., 2014). Empirical studies by Liu et al. (2023) and Wang et al. (2018) identified key dimensions, including openness to admitting mistakes, valuing others’ strengths, a willingness to learn, and a focus on collective success. Creativity on the other hand is defined as the process of idea exploration, generation, championing, and implementation (Scott and Bruce, 1994; Carmeli et al., 2006). While according to Janssen and Giebels (2013), the creative performance of employee is consist of idea generation, idea promotion, and idea realization.

These positive leadership traits/styles are some of the key factors that may foster creativity (Gilmore et al., 2013; Liu et al., 2023). Although research to date has examined the links between several leadership styles and creativity (Carmeli and Paulus, 2015; De Clercq and Mustafa, 2023), but still there is a lack of empirical evidence regarding the relationship of humble leadership with positive outcomes like employee creativity in service sector organizations and its indirect relationship (the mediation effect) on creative performance.

In the past, HL has appeared as a compelling area of study within organizational behavior, with theoretical investigations flaking light on its conceptual foundations and implications for workplace dynamics. Owens and Hekman (2012) proposed a theoretical model that re-conceptualizes leadership as a process of guidance that involves both leader humility and follower response. The model emphasizes the importance of leader humility in creating a positive working organizational climate, promoting team learning, and fostering employee engagement. Skakon et al. (2010) also contribute to the theoretical understanding of HL by exploring its role in charismatic leadership. They argue that humility, when combined into charismatic leadership, improves the follower perceptions of leader sincerity and authenticity, ultimately contributing to positive organizational outcomes. In the context of team dynamics, Owens and Hekman (2016) presented a theoretical framework highlighting the contagion effects of leader humility within teams. Their model suggests that HL positively influence team performance by promoting a collective focus on growth and development. Building on the socialized charismatic leadership perspective. Another study by Owens et al. (2011) offer a theoretical exploration of humility in organizations, discussing its relevance and implications. Their conceptual analysis highlights the potential impact of HL on employee attitudes and behaviors, providing a foundation for further empirical investigations.

In the past studies, HL has appeared as a fundamental aspect of effective organizational management, emphasizing collaboration, openness, and a genuine concern for the well-being of team members (Owens and Hekman, 2016). Simultaneously, CP stands as a critical determinant of an organization’s ability to innovate and adapt in today’s dynamic business environment (Shalley and Perry-Smith, 2008). HL also promotes employees’ engagement and resilience, which can lead to increased job satisfaction and creativity (Liu et al., 2023). Empirical investigations underscore the positive influences of humble leadership on employee outcomes. Owens et al. (2013) found that humble leadership positively predicts engagement, job satisfaction, and performance. The mechanisms include cultivating a supportive work environment, building trust, and enhancing communication within the team (Owens et al., 2013; Owens and Hekman, 2016). Adding more to empirical studies by Chen H. et al. (2021) demonstrated that humble leadership positively influences employee proactive behavior and performance through the promotion of psychological safety and need satisfaction. Not only has this but HL in existing research also explored the motivational perspective of humble leadership. Ou et al. (2014) and Walters and Diab (2016) investigated the effects of humble leadership on employee outcomes and identified humility as a critical factor in fostering employee intrinsic motivation, psychological safety, and engagement. This study, along with others, contributes to understanding how humble leadership aligns with motivational theories and impacts employee motivation positively. As mentioned that HL is considered to be one of the vital element for employee positive outcome, the evidences can be traced in number of empirical studies like, HL has been investigated to gage the project success, where HL can provide valuable input to the get the project success (Ali et al., 2020, 2021). Not only this but HL is also considered to be one of the key factors in influencing the employee career success (Chughtai and Arifeen, 2023), work engagement (Abbas et al., 2021), psychological empowerment (Ali et al., 2020), and employee’s emotional and ethical behavior (Naseer et al., 2020).

In addition, leaders who exhibit humility are more likely to create a positive work environment. However, while the separate links between humble leadership (HL), intrinsic motivation (IM), work engagement (WE), and creative performance (CP) have been explored (Al Hawamdeh, 2022; Al Hawamdeh and AL-edenat, 2022; Zheng and Ahmed, 2022; Liu et al., 2023), but on the other hand prior studies have suggested that HL’s effect on performance is not only direct; instead, other variables mediate it (Janssen and Giebels, 2013). Therefore; a notable gap exists in understanding the nuanced interplay among the above mentioned variables. Specifically, the literature lacks a comprehensive investigation into the mediation and serial mediation effects of intrinsic motivation (relatedness, competence, and autonomy; Amabile et al., 1994; Ryan and Deci, 2000; Deci et al., 2017) and work engagement (vigor, dedication, and absorption at work place; Schaufeli, 2017) in the relationship between humble leadership (a bottom up approach of acknowledging limitations and mistakes, recognizing followers’ strengths and contributions, and modeling teachability; Owens and Hekman, 2012) and creative performance (idea exploration, generation, championing, and implementation; Scott and Bruce, 1994; Carmeli et al., 2006). Therefore, the aim of this study is to address the above mentioned gap by providing a comprehensive understanding both empirically and theoretically.

This study seeks to integrate the research model, consisting of humble leadership, creative performance, intrinsic motivation, and work engagement, within the context of the Job Demands-Resources (JD-R) model and Self-Determination Theory (SDT). The JD-R model provides a comprehensive framework for understanding the impact of job demands and resources on employee well-being and performance. Concurrently, SDT offers insights into the role of intrinsic motivation and self-determination in fostering optimal functioning and creativity. By combining these theoretical perspectives, we aim to explore the intricate relationships among these variables and contribute to a deeper understanding of how HL influences CP through the mediating mechanisms of IM and WE.

On the other hand, the JD-R model posits that job demands and resources influence employee well-being and performance. In our research model, humble leadership serves as a key job resource, potentially mitigating job demands and fostering a positive work environment. Humble leaders are likely to create a supportive and empowering atmosphere, reducing job stressors, and enhancing employees’ overall job satisfaction. This positive influence of humble leadership aligns with the JD-R model. The JD-R model (Bakker et al., 2007) provides a framework for understanding the impact of job resources on employee well-being and performance. Humble leadership, as a job resource, is theorized to positively influence work engagement and, subsequently, creative performance.

Self-Determination Theory emphasizes the importance of intrinsic motivation and autonomy support in promoting individuals’ psychological well-being and performance. Intrinsic motivation, identified as a mediator in our model, is expected to play a crucial role. Humble leaders, by acknowledging and appreciating employees’ contributions, may enhance intrinsic motivation, leading to a more engaged and creative workforce. This integration allows us to explore how humble leadership, as a form of autonomy support, aligns with SDT principles and contributes to the fulfillment of basic psychological needs, fostering intrinsic motivation among employees. To elucidate the underlying processes through which humble leadership influences creative performance. By examining these mediating mechanisms within the JD-R framework and SDT, we aim to uncover the nuanced pathways that link humble leadership practices to enhanced creative performance. Understanding these processes is crucial for both theoretical development and practical implications, providing insights into how organizations can cultivate a work environment that nurtures creativity through leadership strategies and motivational factors.

Using insights from past studies showing that HL favorable outcomes such as higher work engagement (Bao et al., 2018), greater job satisfaction and happiness (Owens and Hekman, 2012), increased job performance, and superior job performance (Rego et al., 2017), this study predicts a positive relationship between employees’ perceptions of leader humility and their creative performance. Further, considering the motivational potential of humble leadership (Carnevale et al., 2019), we argue that leader’s humility will positively influence employees’ intrinsic motivation. Similarly, consistent with past research findings showing that leaders’ humility increases employee dedication and enthusiasm for work (Bakker, 2022), a positive relationship is predicted between humble leadership and work engagement. Finally, this study postulates that intrinsic motivation and work engagement will individually as well as sequentially mediate the association between humble leadership and creative performance. In particular, it is assumed that employees’ positive perceptions of leader humility will increase their intrinsic motivation and work engagement, resulting in greater creative performance. These postulations are congruent with the motivation process of the job demands-resources model and self-determination theory suggesting that the abundance of job resources (positive aspects of jobs such as humble leadership) often leads to desirable work outcomes (e.g., higher levels of creativity, innovation, and productivity) through their positive impacts on employee motivation and work engagement (Ou et al., 2014; Chen et al., 2020; Sharif et al., 2021). The choice of HL as an independent variable and creative performance as dependent variable is congruent with the past researches (Chen L. et al., 2021; Kim, 2022; Liu et al., 2022; Kelemen et al., 2023) also our choice of mediation can be found in the past studies (Ryan and Deci, 2019; Urban and Urban, 2023).

**Figure 1:**
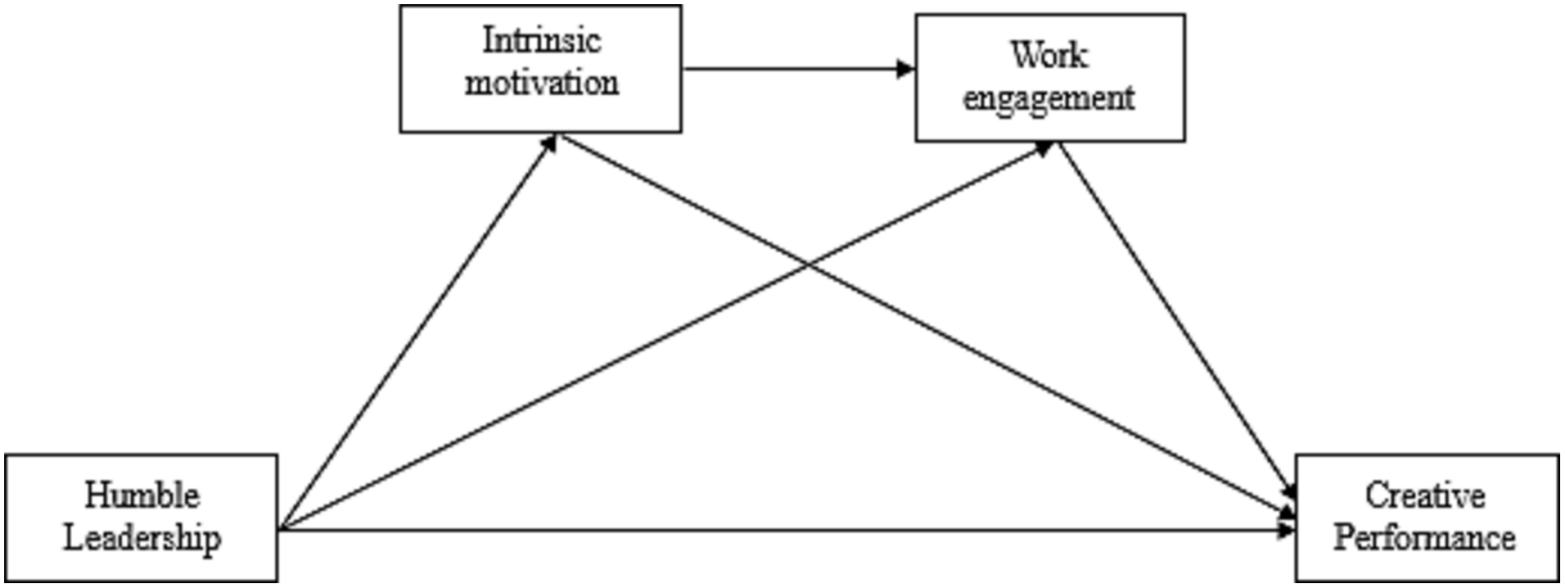
Conceptual model.

This study contributes in the following ways: first, while studies examining the links between HL and employee performance abound (Rego et al., 2021), this study is among very few empirical works ascertaining the association between HL and CP; second, this study explicates how HL can culminate into increased CP. Third by incorporating Self-Determination Theory (SDT) and the Job Demands-Resources (JD-R) model, this study aim to contribute to the advancement of theoretical frameworks in organizational psychology. It will extend these theories by exploring how IM and WE, individually and serially, mediate the relationship between HL and CP. Finally the examination of serial mediation involving IM and WE represents a novel contribution. This sequential analysis will add depth to the understanding of the underlying processes, shedding light on the intricate ways in which these variables may interact and influence creative performance over time. In summary, not only does this study broaden our understanding of how employees’ positive perceptions of leader humility impact their creative performance, but it also offers valuable insights for management practice. The research model has been shown in Figure 1.

## 2 Theory and hypotheses

### 2.1 Humble leadership and creative performance

Humility entails analyzing oneself fairly and accurately, appreciating the strengths of followers, and learning from others (Owens et al., 2013). Past researches have shown that leader humility can have profound effects on employees and the organization. For instance, humility of a leader can increase followers’ engagement with work (Owens and Hekman, 2016), commitment to the organization and job (Basford et al., 2014), and decrease certain negative attitudes and detrimental behaviors (Owens and Hekman, 2016). This is mainly because humble leaders follow an employee-centered approach, i.e., they build/develop congenial interpersonal relationship with their followers, resulting in positive outcomes. In keeping with these findings, we contend that HL can play important role in fostering CP of their followers. We argue this based on the positive attributes of humble leadership. In a study by Ou et al. (2017), humble leadership was found to be positively related to employee learning orientation. A learning-oriented culture supports continuous improvement and innovation, contributing to enhanced creative performance. On the other hand, followers may feel enthused to show superior performance, introduce new ideas, and provide creative and out of the box solutions to the current problems while working with humble leaders (Min et al., 2007). Generating novel ideas and providing creative solutions to the current problems entails risks, i.e., all novel ideas are not be successful or accepted by everyone, leading one to feel discouraged and frustrated. However, humble behaviors (e.g., acknowledging and teachability) of a leader may steer the followers away from such negative feelings and emotions, and invoke a sense of gratitude that may prevent them to get off-tracked (Owens and Hekman, 2012).

Creativity often arises in situations that are ill-defined/unstructured and complex; therefore, one may encounter substantial challenges during the idea generation process. A humble leader can help the followers to off-set such challenges, i.e., he/she may engage in activities and teach the followers how to overcome hurdles (Carmeli et al., 2013). This may encourage followers to exert more effort and energy to engender and implement creative ideas. Therefore, it may be assumed that HL can positively influence followers’ CP (Zhou and Wu, 2018). This notion is also consistent with the JD-R model, which suggests that favorable job-related factors (e.g., humble leadership) can facilitate important outcomes (e.g., superior performance) (Owens et al., 2013). Therefore, the following hypothesis is proposed:

H1: Humble leadership will be positively related to creative performance.

### 2.2 Humble leadership and intrinsic motivation

Motivation is a major psychological force that drives one to act and behave in certain ways (Waterhouse et al., 2014; Jensen and Bro, 2018). There are two main types of motivation: intrinsic motivation (a drive that comes from within) and extrinsic motivation (a drive that revolves around external factors) (Chen and Hsieh, 2015). Although both types of motivation are equally important, this study is focused on intrinsic motivation because it has been argued to be a major driving force of employees’ effort, performance, and creativity (Park and Word, 2012; Chen and Hsieh, 2015; Demircioglu and Chen, 2019; Mussagulova and Demircioglu, 2022). Employees are motivated by three basic psychological needs as stated by Self-Determination Theory (SDT) these needs are autonomy in their job, relatedness, and competence (Deci et al., 2017). When these prerequisites are met, employees experience a sense of meaning and self-determination, which in turn, fosters intrinsic motivation and their ability to respond to work demands and overcome challenges (Mussagulova and Demircioglu, 2022). The ability of a supervisor/leader to build a supportive working environment redirects the concern for employees’ feelings and needs, which helps to solve work-related problems and increases employees’ self-determination and interest for the work (Deci et al., 2017).

Notwithstanding the fact that intrinsic motivation comes purely from within, we argue that leaders’ humility can bolster/strengthen/reinforce it. In particular, we propose that individuals working under the guidance of a humble leader will have a stronger intrinsic motivation. We argue this based on the following reasons. First, HLs support their followers’ distinctive capabilities, telling and helping them to recognize the importance of their contributions; thus boosting their sense of meaning and competence in the work (some of the fundamental conditions for intrinsic motivation). Second, humble leaders are open-minded and have a desire to learn from their followers, leading to frequent communications. The frequent communication and exchange of information builds a stronger sense of connection and affiliation among followers, satisfying their need for relatedness (a driver of intrinsic motivation). Third, HLs willingly accept their own shortcomings and appreciate the strengths of their followers. They belief in their followers’ competencies and are keen to grant autonomy and power to the followers, thus raising their sense of self-determination. In summary, interactions with a humble leader satisfy employees’ psychological needs for competence, relatedness, and autonomy within the organization, thereby bolstering/reinforcing their intrinsic motivation. Therefore, we posit that:

H2: Humble leadership will be positively related to intrinsic motivation.

### 2.3 Humble leadership and work engagement

Work engagement is a positive work-related attitude categorized by dedication (the positive feelings in the form of personal growth, competence, and significance of work), absorption (attachment of employees with their work where they enjoy to spend more time), and vigor (the level of energy and resilience at work; Jeung and Yoon, 2016; Chen et al., 2020). We argue that leader’s humility can foster this attitude. It is a well-established fact that employees engage more in positive activities when they see their leaders demonstrate humility. A humble leader has the capability to form an environment where the subordinates feel comfortable and perform their duties without any fear of undesirable consequences. Admitting his/her own weaknesses and shortcomings and appreciating the strengths of the followers may also result in greater WE (Rego et al., 2019). Researches in the past show that humble leaders can reinforce employee orientation toward learning, job satisfaction, and work engagement (Moss and Ritossa, 2007; Rego et al., 2019; Juyumaya and Torres, 2023). This is mainly because a humble leader gives value and respect to his/her followers; consequently they show greater work engagement. Further, humble leaders play a vital role in building a safe working environment that promotes maximum performance and engagement (Walters and Diab, 2016; Liu et al., 2023). Compared with other leadership styles, humble leaders tend to be more helpful and supportive toward their followers, thus increasing their dedication, absorption, and vigor. According to a study (Juyumaya and Torres, 2023), higher the humble behavior of a leader, higher will be the work related energy and engagement of the workforce. Therefore, a positive relationship can be anticipated between humble leadership and work engagement. This notion is also consistent with the theoretical premise of the JD-R model that favorable aspects of the work (e.g., humble leadership) produce favorable outcomes (e.g., work engagement; Kahn, 1990) and also the JD-R model posits that work characteristics can be classified into two categories: job demands and job resources. Job resources, such as humble leadership in our case, can positively influence work engagement. Humble leadership, by acknowledging and supporting employees, acts as a job resource that enhances work engagement (González-Romá et al., 2006). Therefore, the following hypothesis is proposed:

H3: Humble leadership will be positively related to work engagement.

### 2.4 Intrinsic motivation and creative performance

Intrinsically motivated individual can produce several favorable outcomes (Shalley and Perry-Smith, 2008) including creative performance (Amabile, 1988). Many studies have found a positive correlation between intrinsic motivation and creative performance (Shalley and Perry-Smith, 2008). For example, a study found that employees who were intrinsically motivated were more likely to generate creative ideas (Zhou and Shalley, 2003). The study by Tu and Lu (2016) reported similar findings, showing that intrinsic motivation boosts creativity. Research indicates that providing employees with autonomy and opportunities for self-expression strengthens intrinsic motivation and increases creative performance. Employees who have more job autonomy and the power to make decisions about their tasks display higher levels of creative performance compared with those with limited autonomy. Job autonomy plays a pivotal role in stimulating workers’ creativity as it aligns with their innate psychological nature (Juyumaya and Torres, 2023). When employees are granted freedom and self-determination, they feel motivated to try new things and generate diverse and innovative ideas. The motivation process of the JD-R model also highlights that motivation can lead to desirable outcomes (Eisenberger et al., 2005). Therefore, we propose that:

H4: Intrinsic motivation will be positively related to creative performance.

### 2.5 Work engagement and creative performance

This study postulates that work engagement can foster creative performance. Research has shown that highly engaged employees often demonstrate superior cognitive processing, which in turn, bolsters creativity (Bakker, 2022). Hence, a positive relationship may be predicted between work engagement and creative performance. Further, WE is often associated with positive affect and emotions such as enthusiasm, joy, and happiness toward work (Abdullah and Al-Abrrow, 2022). These measures of positive affect are strongly linked to heightened creativity, as they enhance cognitive processes and promote divergent thinking. Put simply, it may be asserted that engaged employees demonstrate greater performance because they experience a favorable mental state that allows them to generate innovative ideas. Therefore, the following hypothesis is proposed:

H5: Work engagement will be positively related to creative performance.

### 2.6 Intrinsic motivation and work engagement

Higher level of employee intrinsic motivation leads to higher level of favorable outcomes. These favorable outcomes include creativity, job satisfaction, and work engagement (Van den Broeck et al., 2016). On the other hand, work engagement is also associated with positive organizational outcomes such as productivity, less absenteeism, and higher job satisfaction (Schaufeli, 2017). As intrinsic motivation is consist psychological factors like autonomy, relatedness, and competence (Bakker, 2022), these factors if satisfied the employees will be more dedicated and engaged in their work. According to Bakker (2022), intrinsically motivated employees are self-drive to accomplish their task. This phenomena of self-determination leads employees toward more dedication, vigor, and absorption. Job autonomy on the other hand is one of the most powerful tool for employees in their work settings. Giving power and get them involved in the decision making process ultimately increase the intrinsic motivation of the workers. This autonomy in work ultimately results in more work engagement (Deci et al., 2017). This recommends that by cultivating the intrinsic motivation an organization can increase the work engagement of employees which ultimately results in job satisfaction, OCB and affective and cognitive trust on both management and organization. It is important for the organization to understand and nurture the environment of intrinsic motivation and work engagement to foster the performance of the employees. Backing by the above arguments we therefor; posit that

H6: Intrinsic motivation will be positively related to work engagement.

### 2.7 The mediation effects

The humble behavior (job resource) of a leader has the potential to influence the individual creative behavior. But there is a lack of understanding of how these behaviors through the underlying mechanism can predict the performance of the employees specifically the creative performance of the followers (Cho and Perry, 2012; Park and Word, 2012). The past researches emphasized on the direct relationship between HL and employee CP (Liu et al., 2023) and overlooked the mediating role of intrinsic motivation. According to Amabile et al. (1994), intrinsically motivated employees are more likely to generate creative ideas. On the other hand, a leader particularly exhibiting humility can foster the self-determination of the followers, which ultimately may result in improved creative performance (Park and Word, 2012). Prior research has indicated a positive correlation between job related resources, including job autonomy and leader support, and individual resources like self-efficacy, intrinsic motivation, and CP (Demerouti et al., 2010). The provision of a creative and innovative working environment has been found to have a positive impact on employees’ sense of control and resilience, thereby serving as an intrinsic motivator for them to confront and overcome challenging situations (Avolio et al., 2009). Leaders who are perceived as positive and humble can foster creative behavior among employees. This is because the display of humility through positive leadership can enhance self-confidence and reduce the fear of unfavorable feedback which may otherwise impede motivation to express novel ideas (Bass, 1985). Companies may raise the intrinsic motivation of their workers toward their job through HL by creating clear guide lines, procedures, and processes that encourage a shared commitment and a mutual receptivity to new ideas.

In the past, transformational (De Clercq and Mustafa, 2023) and transactional leadership (Juyumaya and Torres, 2023) has been the focal point of studies to predict creativity but the study of HL and its impact on employee creativity has been ignored specially its impact through mediating mechanism of intrinsic motivation (Mussagulova and Demircioglu, 2022). To address this gap we posit that,

H7: Intrinsic motivation will mediate the relationship between humble leadership and creative performance.

The fast paced technological advancements require long-term organizational growth and creative performance, which involves creating innovative goods, services, and procedures (Tierney and Farmer, 2011). Employee engagement is important in this regard due to their motivation to exert more efforts to accomplish the tasks and to give positive output. On the other hand, disengaged employees become the hurdle for achieving excellence in performance and organizational growth (Schaufeli et al., 2006). In contrast to other leadership styles, HL adopts an employee-centered approach to inspire and incentivize their subordinates to enhance the anticipated performance levels for the betterment of the organization. Bass (1985) proposed that a HL who provides guidance, motivation, and stimulates trust can lead to increased employee effort. Such traits indicate that leaders who exhibit humility have the potential to elevate WE levels through their willingness to embrace new ideas and openness to learning, ultimately resulting in improved (CP). According to the Job Demands-Resources (JD-R) theory, employees who have a high level of engagement are likely to demonstrate increased levels of vigor, dedication, and absorption in their job-related duties (Juyumaya and Torres, 2023). Therefore, the humility of a leader assumes an essential part in stimulating the attitudes of employees, including higher levels of energy, dedication, and commitment toward accomplishing tasks (Schaufeli et al., 2006).

The majority of scholarly investigations pertaining to HL procedures focus on models that establish a relationship between leaders’ attributes and their performance, while overlooking the potential mediating influence of WE. Although we have sufficient evidence about the positive relationship of WE with servant leadership (Haar et al., 2017) and transformational leadership (Kovjanic et al., 2013) but the relationship between HL and WE and its mediating role between the indirect relationship of HL and CP is still lacking. Therefore, this study will address this gap. To fill this gap in the literature and theory, we hypothesize that,

H8: Work engagement will mediate the relationship between humble leadership and creative performance.

The past researches have consistently reported a positive relationships between different positive outcomes in the form of work engagement, and other factors, like leadership behavior, job satisfaction, personal growth, job autonomy, and creative performance (Demerouti et al., 2010). Providing a conducive environment to encourage the creative performance has been the subject of many studies (Zhu et al., 2018; Ilha Villanova and Pina e Cunha, 2021; Juyumaya and Torres, 2023). CP has been linking through different mechanism such as WE and IM to overcome the challenges that may hinder the progress of employees as well as organization (Avolio et al., 2009). Humble leadership in this connection plays a crucial role in fostering the psychological encouragement, organizational learning through experimentation of new and innovative ideas. By displaying humble behavior, these leaders can boost motivation level as well as the providing supportive environment for the employee engagement (Deci et al., 2017), which as a result may affect the creative performance. The intrinsic motivation and work engagement can be enhanced by implementing the humble leadership practices like acknowledging limitations and mistakes, recognizing followers’ strengths and contributions, and modeling teachability which may ultimately increase the CP. Although we have enough literature regarding the direct relationship of HL, WE IM, and CP, but the mediating especially the sequential mediating mechanism is totally overlooked over the years. To address this gap, this study will test whether the sequential mediation mechanism works in the indirect relationship between HL and CP or not. Therefore; we posit that,

H9: Intrinsic motivation and work engagement sequentially mediate the positive relationship between humble leadership and creative performance.

## 3 Methodology

### 3.1 Research context, sample, and data collection

Convenience sampling method was used to collect the data from the rapidly growing service sector organizations (telecommunication companies) in Pakistan for the following reasons. The telecom industry frequently faces disruptions, such as the emergence of new communication technologies, changes in consumer preferences, and regulatory shifts. Creative performance is essential for organizations to adapt swiftly and effectively to these disruptions, turning challenges into opportunities. And the ongoing digital transformation in the telecom sector requires creative solutions for adopting new technologies, optimizing operational processes, and ensuring a seamless transition to the digital landscape. Creative performance is instrumental in shaping digital strategies and facilitating organizational change. In the context of Pakistan, leadership styles are frequently shaped by cultural values such as collectivism, respect for authority, and the significance of interpersonal relationships (Sarfraz et al., 2022). Humble leadership, which underscores cooperation and recognizing the contributions of others, aligns effectively with these cultural values. Research suggests that leadership styles underlining humility and interpersonal harmony are more likely to yield positive outcomes in Pakistani organizations (Khokhar et al., 2023). Several studies in the field of humble leadership (HL) have investigated its positive impact on project success in the Pakistani context (Ali et al., 2020, 2021; Waseem et al., 2023). Furthermore, studies in the same context have explored the role of HL in influencing career success, employee creativity, follower emotions, ethical behavior, and work engagement (Abbas and Wu, 2019; Kausar, 2020; Naseer et al., 2020; Abbas et al., 2021). These existing literatures provide a solid foundation for conducting our study within this specified context. And most importantly, the telecom sector has been a “less-researched” area in the OB/HRM research.

To increase the generalizability of this study’s findings, we collected data from two provincial capitals (Karachi and Quetta). The researchers visited the designated head offices and their sub branches (franchises) several times before the formal data collection to obtain authorities’ permission for data collection. The participants were then approached and informed of the nature and purpose of this study and other aspects (e.g., voluntary participation and data confidentiality).

To get most accurate responses and minimize the selection bias for our survey, the researchers visited 12 offices in telecom sector to get the preliminary information about the education, age, gender, working experience, and firm size. The data were gathered in two phases. Phase 1 comprised collecting data from employees while phase two involved obtaining supervisors’ ratings of creative performance. Each supervisor was asked to rate the creative performance of all employees working under his/her supervision. Data were collected in 6 months.

We distributed 780 survey questionnaires among the employees of four major telecom companies. We collected 468 surveys in phase 1, indicating an initial response rate of 60%. After careful examination, 118 surveys were discarded for incomplete information or same responses to all questions. 350 (45%) responses were retained for final analysis, satisfying the following sample size criteria:

1. *n* should be 100 if a model comprises ≤ five variables;
2. the measurement items for each variable are not less than 3; and
3. the communalities of the items are not less than 0.60 (Hair et al., 2006; Hair and Black, 2010).

The demographic profile section of the survey obtained information for the following variables: age, gender, experience, and marital status. 141 (40.3%) employees were aged between 20 and 30 years. 181 (51.7%) were aged between 31 and 40 years. 23 (6.6%) were aged between 41 and 50 years while 5 (1.4%) were aged above 50 years. The gender-wise categorization of the sample was as follows: 218 (62.3%) male and 132 (37.7%) females. 182 (52%) respondents had a working experience of 105 years; 134 (38.3%) respondents had a working experience of 6–10 years; 33 (9.4%) has a working experience of 11–15 years; and only one respondent (0.3%) had a working experience of more than 15 years. Of the 350 respondents, 187 (53%) were unmarried, 159 were (45%) married while 4 (1%) were divorced/widowed.

### 3.2 Measures

Previously validated and developed scales were adopted to measure the constructs using a five point liker scale which ranged from 1 (strongly disagree) to 5 (strongly agree).

#### 3.2.1 Humble leadership

Respondents’ perceptions of humble leadership were measured using a nine-item scale (Ou et al., 2014), with sample items such as, “My leader actively seeks feedback, even if it is critical.” The Cronbach alpha of the nine-item was 0.952. The confirmatory factor analysis (CFA) of the scale showed an excellent fit with the data: ꭓ^2^/*df = 1.344 NFI = 0.992, TLI = 0.996, CFI = 0.998*, and *RMSEA = 0.031* with mean of the scale *0.83 all > 0.75*. The communalities of the items were raged from 0.659 to 0.788.

#### 3.2.2 Creative performance

A nine-item scale (Jensen and Bro, 2018) was used to obtain employees’ ratings of creative performance. The items included, for example, (e.g., “This employee creates new ideas for difficult issues”). The Cronbach alpha of the scale was 0.967. The CFA of the scale showed a good fit: ꭓ^2^/*df = 2.181, NFI = 0.991, TLI = 0.990, CFI = 0.995*, and *RMSEA = 0.058* with mean of the scale *0.84 all > 0.82*. The communalities of the items ranged from 0.753 to 0.834.

#### 3.2.3 Intrinsic motivation

Five items “I enjoy finding solutions to complex problems at work” by Tierney et al. (1999) were used to measure the intrinsic motivation of employees. The Cronbach alpha of the scale was 0.959. The CFA of the scale showed good results: ꭓ^2^/*df = 1.629, NFI = 0.995, TLI = 0.996, CFI = 0.998*, and *RMSEA = 0.042*. The communalities of the items ranged from 0.784 to 0.897.

#### 3.2.4 Work engagement

Respondents’ work engagement was measured using a nine-item scale and included “At my work, I feel bursting with energy” (Schaufeli et al., 2006). The Cronbach alpha of the nine-item scale was 0.969. The CFA of the scale showed good results: ꭓ^2^/*df = 1.735, NFI = 0.993, TLI = 0.993, CFI = 0.997*, and *RMSEA = 0.046* with mean of the scale *0.87 all > 0.82*. The communalities of the items ranged from 0.736 to 0.844.

## 4 Results

The summary of CFA results is shown in Table 1. The CFA is one of the multivariate statistical tools used to measure goodness of fit. In particular, this technique is used to check how well a particular scale represents a variable. Therefore; we initiated our analysis by running the CFA through AMOS. Results revealed that all the scales and hypothesized model fitted the data well as compare to alternative models (One factor, two factor, and three factor models).

**Table 1:** Confirmatory factor analysis.

Models	ꭓ^2^/df	NFI	TLI	CFI	RMSEA
One factor model	10.8	0.66	0.64	0.68	0.16
Two factor model IM + WE,HL + CP	7.58	0.76	0.76	0.78	0.13
Two factor model CP + IM,HL + WE	7.55	0.72	0.75	0.81	0.13
Two factor model HL + IM,WE + CP	6.41	0.8	0.81	0.83	0.12
Three facto model CP + HL,IM,WE	6.12	0.8	0.81	0.83	0.12
Three factor model HL + IM,WE,CP	4.32	0.86	0.88	0.89	0.09
Three factor model IM + WE,CP,HL	3.89	0.87	0.89	0.9	0.91
Four factor hypothesized model	1.71	0.948	0.974	0.978	0.045

χ^2^, Chi-square test; df, Degrees of freedom; NFI, Normed fit index; TLI, Tucker Lewis index; CFI, Comparative fit index; and RMSEA, Root mean square error of approximation.

Table 2 represents the reliability, validity, and correlations among the variables. The results satisfied the following criteria: composite reliability (CR) ≥ 0.7, Average variance extraction (AVE) ≥ 0.50, and Maximum shared variance (MSV) < AVE (Fornell and Larcker, 1981).

**Table 2:** Reliability, validity, and the correlations among the variables.

	CR	AVE	MSV	MaxR(H)	HL	WE	CP	IM
HL	0.952	0.690	0.260	0.955	**0.831**			
WE	0.967	0.766	0.410	0.969	0.402^**^	**0.875**		
CP	0.959	0.725	0.419	0.961	0.509^**^	0.583^**^	**0.851**	
IM	0.960	0.828	0.419	0.964	0.510^**^	0.640^**^	0.647^**^	**0.910**

CR, Composite reliability; AVE, Average variance extracted; MSV, Maximum shared variance; and MaxR(H), Maximum reliability. The square roots of average variance extracted (AVE) are given in bold figures diagonally for each variable. ∗∗*p* < 0.01.

To detect common method bias (CMB), Herman’s single factor test was run using exploratory factor analysis (EFA) and CFA. The single factor analysis did not show a serious concern as the variance explained by a single did not exceed 50%.

Table 3 represents the summary of hypotheses testing (1–6) results. Different combinations of linear and multiple regression using the PROCESS macro for SPSS were used to test the hypothesized relationships. The results regarding hypothesis 1 revealed that the total effect of humble leadership on creative performance was significant positive (*β* = 0.466, *p* < 0.001, LLCI = 0.421, ULCI = 0.631), supporting hypothesis 1. Similarly, the total effect of humble leadership on intrinsic motivation was significant positive (*β* = 0.483, *p* < 0.001, LLCI = 0.481, ULCI = 0.709), supporting hypothesis 2. The results further revealed a statistically significant relationship between humble leadership and work engagement (*β* = 0.378, *p* < 0.001, LLCI = 0.280, ULCI = 0.475), supporting hypothesis 3. Further, hypothesis was also supported in that the path linking intrinsic motivation to creative performance was statistically significant (*β* = 0.606, *p* < 0.001, LLCI = 0.478, ULCI = 0.632). The total effect of work engagement on creative performance was significantly positive (*β* = 0.547, *p* < 0.001, LLCI = 0.518, ULCI = 0.717), thus hypothesis 5 was also supported. Further, the association between intrinsic motivation and work engagement was statistically significant (*β* = 0.612, *p* < 0.001, LLCI = 0.429, ULCI = 0.564), supporting hypothesis 6.

**Table 3:** Regression analysis.

Relationship	β	*t*	Sig.	*R* ^2^	*F*	Sig.	Hypotheses
H1: HL− > CP	0.466	9.831	0.000	0.217	96.646	0.000	Supported
H2: HL− > IM	0.483	10.288	0.000	0.233	105.848	0.000	Supported
H3: HL− > WE	0.378	7.612	0.000	0.143	57.938	0.000	Supported
H4: IM− > CP	0.606	14.215	0.000	0.367	202.053	0.000	Supported
H5: WE− > CP	0.547	12.194	0.000	0.299	148.701	0.000	Supported
H6: IM− > WE	0.612	14.454	0.000	0.375	208.905	0.000	Supported

HL, Humble leadership; CP, Creative performance; IM, Intrinsic motivation; and WE, Work engagement. ^***^p < 0.001.

The total and direct and indirect effects of humble leadership intrinsic motivation and work engagement (mediators) on creative performance are shown in Table 4. The direct effect of humble leadership on creative performance was partially mediated by intrinsic motivation (*β* = 0.2553, *p* < 0.001, LLCI = 0.1505, ULCI = 0.3601) as the *β* value was less than the value of the total effect. The mediation effect of IM (Indirect effect) was also significant (*β* = 0.2707, *p* < 0.001, LLCI = 0.2042, ULCI = 0.3494). Thus supporting hypothesis 7. Moving toward hypothesis 8, the direct effect of humble leadership on creative performance was also partially mediated by work engagement (*β* = 0.3415, *p* < 0.001, LLCI = 0.2401, ULCI = 0.4430) because the *β* value was less than the value of the total effect. The mediation effect was also significant (*β* = 0.1845, *p* < 0.001, LLCI = 0.1250, ULCI = 0.2518). Moreover; the absence of non-zero between LLCI and ULCI also proves that the relationship is significant. Therefore, the above empirical evidences support our hypothesis.

**Table 4:** Mediation analysis.

Relationship	TE	DE	IE	SE	LLCI	ULCI	Remarks
H7: HL− > IM− > CP	0.526	0.2553	0.2707	0.0371	0.2042	0.3494	Partial mediation
H8: HL− > WE− > CP		0.3415	0.1845	0.0327	0.125	0.2518	Partial mediation

TE, Total effect; DE, Direct effect; IE, Indirect effect; LLCI, Lower level confidence interval; and ULCI, Upper level confidence interval.

Table 5 shows the total direct and indirect effects of humble leadership on creative performance mediated by intrinsic motivation and work engagement.

**Table 5:** Serial mediation analysis.

Effects of HL on CP	Effect	SE	t	LLCI	ULCI	Conclusion
Total effect (HL − > CP)	0.526	0.0535	9.8309	0.4208	0.6312	
Direct effect (HL − > CP)	0.224	0.0519	4.3267	0.1224	0.3265	
Indirect effect III (HL− > IM− > WE− > CP)	0.0781	0.0214	3.64	0.0369	0.1215	Partial serial mediation

**Figure 2:**
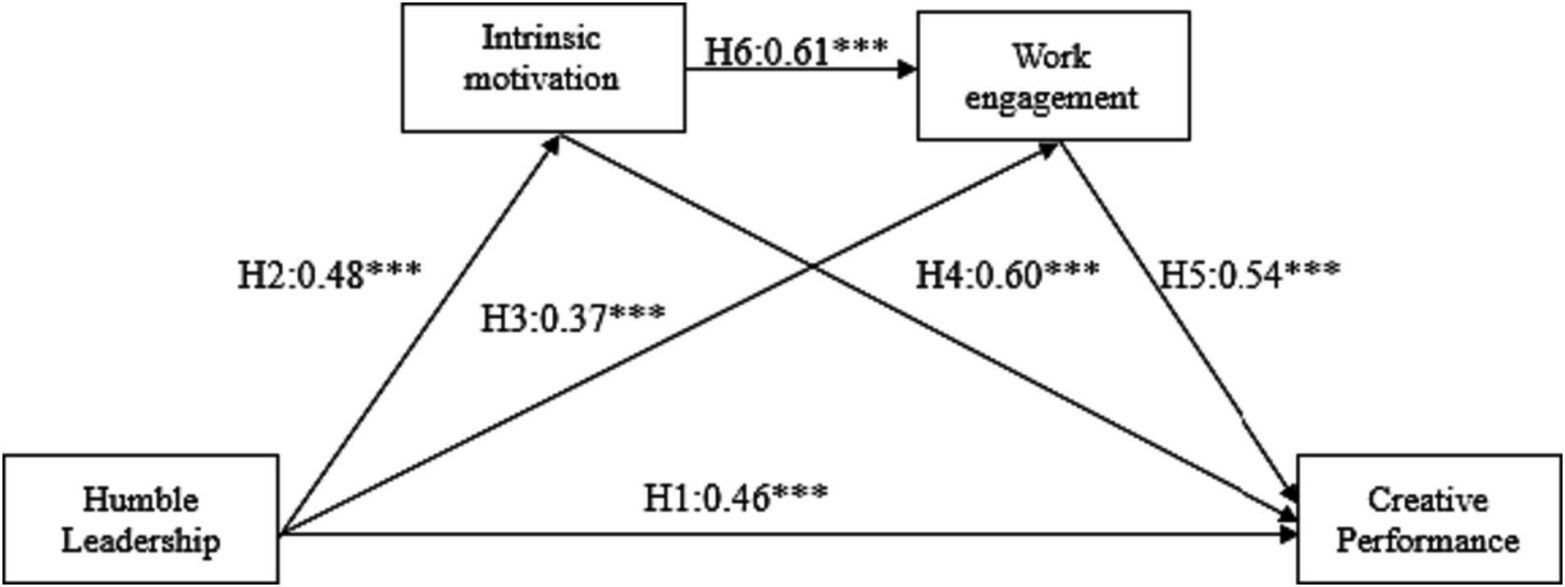
Main effects, *n* = 350, ^***^p < 0.001.

The study assessed the serial mediation with intrinsic motivation and work engagement serially mediating the relationship between humble leadership and creative performance. The results revealed a significant indirect effect of humble leadership on creative performance through intrinsic motivation and work engagement (*b* = 0.0781, *t* = 3.64), supporting H9. Furthermore; the direct effect of humble leadership on creative performance in presence of the mediators was also found significant (*b* = 0.224, *p* < 0.001). Hence, there is a partial serial mediation of intrinsic motivation and work engagement on the relationship between humble leadership and creative performance. The summary of the serial mediation results is presented in Table 5. While Figure 2 represents the path-coefficients (*β*).

## 5 Discussion

Humility is considered to be one of the fundamental features that leaders need to display in this ever changing global competitive and complex business environment. Therefore, the demand for research on leader humility has been growing, especially for the service sector organizations (Luu, 2021). Responding to such calls for research, this study investigated how leader humility affects followers’ creativity. In particular, this study examined the humble leadership-creative performance link, taking into account the mediating roles of IM and WE using STD and the JD-R model as theoretical lens.

The study contributes to the leadership and creativity literature in several ways. This study provides an empirical evidence about the relationship between HL and CP (Mumford and Fried, 2014). It also confirms the speculative statements about the importance of leadership behavior in organizational context by supporting the underlying mechanism of intrinsic motivation and work engagement both individually and sequentially. The empirical evidence from this study furthers our understanding regarding the connection between leader’s humility and employee outcomes. Humble leadership was found to be positively associated with creative performance, intrinsic motivation, and work engagement. Although these relationship were examined and established in previous researches (Woods et al., 2018; Zheng and Ahmed, 2022; De Clercq and Mustafa, 2023; Juyumaya and Torres, 2023; Liu et al., 2023) but the role of IM and WE (as mediators) needed a call for further investigation. The underlying mechanism through which leader humility can influence creative performance remain largely unknown (Gilmore et al., 2013). To address this gap in theory and literature, the present study provides empirical evidence in the form mediating role of IM and WE. The findings of this study are consistent with past research (Shalley and Perry-Smith, 2008) as it highlights the importance of psychological factors in linking humble leadership with performance.

The present study considerably contributes to the existing literature by evolving theoretical frameworks in organizational psychology, mainly in the realms of humble leadership (HL), creative performance (CP), and the mediation processes of intrinsic motivation (IM) and work engagement (WE). The theoretical basis of this research draw from Self-Determination Theory (SDT) and the Job Demands-Resources (JD-R) model, providing a robust framework for understanding the nuanced relationships among these variables.

While several studies have discovered the link between humble leadership and various employee outcomes (Abbas and Wu, 2019; Kausar, 2020; Ali et al., 2021; Waseem et al., 2023), our study is one of the few empirical works explicitly examining the association between humble leadership and creative performance (CP). By empirically establishing this relationship, our research extends the understanding of how humble leadership, with its focus on collaboration, openness, and acknowledging contributions, can positively influence employees’ creative performance. This empirical evidence adds depth to the existing theoretical discussions on humble leadership’s impact on organizational dynamics.

Integrating SDT into our research design allows us to investigate into the motivational processes underlying the relationship between humble leadership and creative performance. By explicitly examining how intrinsic motivation (IM) mediates this relationship, our study advances SDT by validating how humble leadership practices add up to employees’ intrinsic motivation, thereby fostering creativity. This exploration aligns with SDT’s emphasis on the importance of autonomy support and intrinsic motivation in promoting psychological well-being and optimal functioning.

Within the JD-R model, humble leadership is conceptualized as a key job resource that mitigates job demands and adds to a positive work environment. Our study extends the JD-R model by empirically testing the role of humble leadership as a resource that not only lessens job stressors but also positively influences creative performance. This expansion aligns with the JD-R model’s focus on the dual impact of resources on both employee well-being and performance.

A notable contribution of our study lies in the examination of sequential mediation involving intrinsic motivation and work engagement. While previous research has examined these variables individually, our study advances the literature by demonstrating how they operate sequentially to mediate the association between humble leadership and creative performance. This sequential analysis provides a more nuanced understanding of the temporal dynamics and intricate ways in which these variables interact over time.

Finally, while most of the humble leadership research has been conducted in the western context, this study is unique in a sense that it examines the role of humble leadership in an under-studied cultural context (Pakistan); thus adding new insights to the leadership literature.

## 6 Implication for theory

Using JD-R model and STD as theoretical lens, our study provides a holistic framework for understanding the relationships among humble leadership, intrinsic motivation, work engagement, and creative performance. The successful application of these theories in a specific context contributes to the broader literature on organizational behavior and leadership. The acceptance of hypotheses regarding humble leadership’s impact on intrinsic motivation, work engagement, and creative performance suggests a sequential mediation pathway consistent with the JD-R model and also consistent to the previous researches (Woods et al., 2018; De Clercq and Mustafa, 2023). This reinforces the idea that job resources, such as humble leadership behaviors, can positively influence employees’ internal motivational processes, leading to enhanced work engagement and creative performance. The study’s findings contribute to the JD-R model by providing empirical support for the role of humble leadership as a job resource. This extends the understanding of job resources beyond traditional factors, demonstrating that leadership behaviors can act as resources that foster employee well-being and positive outcomes.

The acceptance of hypotheses related to intrinsic motivation aligns with SDT, which posits that individuals have innate psychological needs for autonomy, competence, and relatedness. Our findings reinforce the idea that humble leadership can contribute to employees’ satisfaction of these needs, thereby enhancing intrinsic motivation. The study’s focus on creative performance as the dependent variable contributes to the literature on leadership and creativity. The positive impact of humble leadership on creative performance suggests that leaders who exhibit humility can create an environment conducive to innovation and idea generation. The study provides a theoretical foundation for leadership development programs. Organizations can leverage the insights from our research to design interventions that cultivate humble leadership qualities, recognizing their potential to positively influence intrinsic motivation, work engagement, and creative performance.

In summary, the study’s theoretical implications lie in the advancement and integration of the JD-R model and SDT, providing a nuanced understanding of how humble leadership influences intrinsic motivation, work engagement, and creative performance in the telecom sector. This knowledge can inform future research and contribute to the development of effective leadership practices and interventions.

## 7 Implications for practice

This study offers following practical implications for the managers and organizations. First, the study emphasizes on the importance of humility of a leader in contemporary business environment where a leader faces challenges to cope up with the turbulent environment (Mao et al., 2019). The humble behavior is beneficial both for the followers and for the organization. To foster this behavior, we recommend integrating humility initiatives into daily management practices (Seidle et al., 2016). Humility can be developed through formal training and development programs. This will encourage the present day’s leadership to better understand the importance of humility in current era because this behavior of a leader can lead to a number of favorable outcomes in the form of self-awareness, personality grooming, respect, knowledge sharing, and willingness to learn. Managers should focus on enhancing intrinsic motivation among employees. Recognize and reward employees for their achievements, provide opportunities for skill development, and encourage a sense of autonomy in tasks. This can contribute to higher levels of intrinsic motivation. Humble leaders should create an open and transparent communication culture within the organization. This involves actively listening to employees’ ideas, concerns, and feedback. A communication-rich environment can enhance intrinsic motivation by making employees feel valued and heard.

The findings of our study show that the impact of humble leadership on creative performance is mediated by intrinsic motivation and work engagement, therefore, these training programs should also focus to fulfill the needs of relatedness, competence, and autonomy (Intrinsic motivation) and it is also evident from the results of our study that if these psychological needs are satisfied the employees show more dedication, vigor, and absorption (work engagement) in work environment. On the other hand, the demands, personality, and way of cognitive processes of every employee are different from others. Therefore; while exhibiting the humility, the leader should learn how to behave in different conditions and with different employees. Because everyone cannot be treated in the same way. If the subordinates display more creativity the leader should encourage him and he should be given more authority in order to make more fruitful contributions to the organization. Moreover; the employees who perform below expectations, should be given more structured and guided objectives to take maximum out of him. Promoting work engagement is crucial for creative performance. Managers can foster engagement by providing challenging tasks, opportunities for skill utilization, and creating a positive work atmosphere. Encourage employees to take ownership of their work, fostering a sense of pride and commitment. Humble leaders should actively recognize and appreciate the contributions of their team members. Acknowledging individual and collective achievements fosters a positive team environment, boosting both intrinsic motivation and work engagement.

Leaders should find a balance between providing employees with autonomy in their work and offering the necessary support. Humble leaders recognize the strengths of their team members and empower them, but they also provide guidance and support when needed. Managers should recognize that individuals may respond differently to leadership styles. Tailor leadership approaches based on the unique needs and preferences of team members to enhance intrinsic motivation and work engagement. Regular feedback and development discussions are vital. Humble leaders should engage in constructive feedback sessions, helping employees understand their strengths and areas for improvement. This contributes to ongoing development and sustained intrinsic motivation. Foster a culture that values continuous learning and innovation. Humble leaders can encourage experimentation, tolerate reasonable risks, and support a learning mindset within the organization, thereby promoting creative performance. Implement mechanisms for monitoring and assessing the progress of initiatives aimed at promoting humble leadership, intrinsic motivation, and work engagement. Regular assessments can help refine strategies and ensure alignment with organizational goals.

Leaders should regularly assess and review employees’ performance to ensure that they are on the right track. Further, from a personnel training perspective, organizations are advised to conduct training programs, including supportive team-building activities, particularly targeting less creative employees. These programs aim to promote their initiative and enhance their value as human capital within the organization. Finally, organizations should provide more training opportunities to develop a more conducive working environment for the employees working there in. These programs will further enhance their capabilities and will further add up in human capital within the organization. These managerial implications highlight the importance of integrating humble leadership into organizational practices while emphasizing intrinsic motivation and work engagement as critical factors in enhancing creative performance.

## 8 Limitations and future research

Like any other study, our study is subject to certain limitations. First, our hypothesize model based on well-established self-determination theory (Deci et al., 2017) and it is aligned with previous researches (Chen and Hsieh, 2015; Rego et al., 2017). But we could not establish a definite causal relationships. The reverse causality may be possible between the relationship of leadership and creativity. It is possible that a leader may be humble for those who are creative and it is also possible that highly creative employees influence the leader to be humble. Therefore; future research can be conducted to overcome this limitation.

Next, the chances of common method bias always exist (Hassan et al., 2015), although we asked the leaders to rate the performance of their followers but the chances of biasness are always present in such evaluations. Our analysis and measurements were based on the perception of leader rather than some objective measures (Rego et al., 2017). Humble leaders may over rate their followers due to their nature of leadership style (Bharanitharan et al., 2021). To authenticate our findings, we recommend future research to use three prong approach to evaluate the followers, i.e., to include the objective records from HR departments, self-report measures, and co-worker evaluations.

This study was conducted in Pakistan and contributes to the literature by verifying the effects of humility in non-western context. Humility is a culturally influenced phenomenon where different cultures have different concepts of humility for example the religious context and other cultural aspects so this study is limited to different cultural contexts. Therefore, we suggest the future research to investigate the humility across cultures both in eastern and western contexts in order to increase the generalizability of the findings. Further research on humble leadership and creative performance can be undertaken by considering additional variables in the form of mediators and moderators such as job satisfaction, other leadership styles, leader’s political skills, effective and cognitive trust, and emotional intelligence etc. (Bharanitharan et al., 2019) Our study used a cross-sectional design, it may also limit the establishment of causal relationships. Longitudinal studies could provide more insight into the temporal nature of the relationships between humble leadership, intrinsic motivation, work engagement, and creative performance. Given that all data were collected from the same respondents, common method bias might be a concern. Respondents might have provided socially desirable responses, potentially inflating the strength of the relationships.

The reliance on self-report measures for variables such as humble leadership, intrinsic motivation, work engagement, and creative performance might introduce response biases. Consider supplementing self-reports with objective performance measures or obtaining multi-source feedback. The telecom sector may have unique characteristics that could impact the study’s generalizability. Future research might explore whether similar findings emerge in other service sectors or industries. The study’s external validity may be limited to the specific organizational and cultural context of the telecom companies in Pakistan. Cautions are recommended to be taken when applying the findings to organizations with different cultural, regulatory, or economic conditions. Our study endorses the positive aspects of humble leadership but being humble does not mean to be inherently good. Future research could explore the circumstances in which leader’s humility may lead from its positive effects to negative effects. Therefore, it is suggested to check the dark side of being too humble in organizational setups.

## 9 Conclusion

Drawing on JD-R model and SDT, this study investigated the impact of humble leadership, intrinsic motivation, and work engagement on creative performance. The results of the collected data reveled that HL is positively and significantly associated with CP, IM, and WE. While IM and WE are also positively associated with each other and CP. Furthermore; IM and WE both individually and serially mediated the positive relationship between HL and CP. The results of mediation were partially mediate it may be because the employees may respond differently to humble leadership, and individual differences in personality, skills, or experiences might influence how intrinsic motivation and work engagement mediate the relationship and the organizational culture, shaped by humble leadership, may have a direct impact on creative performance. In some cases, the culture itself, rather than intrinsic motivation and work engagement, may act as the primary driver of creativity. Some employees may be naturally more intrinsically motivated or engaged, affecting the mediation process. In conclusion, our study highlights the importance of two mediators. These mediators can influence the relationship between HL and CP. Therefore, the importance of these underlying mechanism cannot be ignored while creating conducive environment for creative performance of employees and organization.

## Data availability statement

The raw data supporting the conclusions of this article will be made available by the authors, without undue reservation.

## Ethics statement

The studies involving humans were approved by Yanshan university ethics committee. The studies were conducted in accordance with the local legislation and institutional requirements. The participants provided their written informed consent to participate in this study.

## Author contributions

HL: Conceptualization, Project administration, Resources, Supervision, Formal analysis, Writing – original draft. SJ: Formal analysis, Methodology, Writing – original draft. MA: Writing – review & editing. AM: Investigation, Validation, Writing – review & editing.
